# The Potential Role of Egg‐Derived Xeno‐miRs in Chemotherapy Response: An In Silico Approach

**DOI:** 10.1002/fsn3.70332

**Published:** 2025-05-23

**Authors:** Berkcan Doğan

**Affiliations:** ^1^ Institute of Health Sciences Department of Translational Medicine Bursa Uludag University Bursa Türkiye; ^2^ Faculty of Medicine Department of Medical Genetics Bursa Uludag University Bursa Türkiye

**Keywords:** chemotherapy response, cross‐kingdom interaction, dietary microRNAs, egg‐derived microRNAs, in silico analyses

## Abstract

Exogenous microRNAs (Xeno‐miRs), primarily derived from dietary sources, are detectable in host biofluids and influence gene expression through cross‐kingdom regulation. Despite growing interest, their impact on human diseases, especially cancer, remains controversial and requires further investigation. However, the specific implications of diet‐derived Xeno‐miRs in chemotherapy response remain largely unexplored. This study assesses the potential functions and possible implications of egg‐derived miRNAs in chemotherapy response with an in silico approach. This study presents the first evaluation of the contribution of diet‐derived miRNAs in modulating chemotherapy outcomes. Egg‐derived miRNAs were retrieved from the Dietary MicroRNA Database, and their human homologs were identified. Target genes and transcription factors were predicted using mirDIP and TransmiR databases, respectively. Pathway enrichment analysis was conducted with DIANA‐miRPath. Expression patterns of Xeno‐miRs were analyzed using the CancerMIRNome database, and differentially expressed target genes were identified using TCGA and GTEx data via GEPIA2. The chemotherapy response of Xeno‐miRs was assessed using ncRNADrug. Fifty‐five egg‐derived Xeno‐miRs were initially retrieved, among which 17 human homologs were further analyzed. Notably, the downregulation of *hsa‐miR‐30a‐5p* and *hsa‐miR‐146a‐5p* was associated with increased sensitivity to fluorouracil and oxaliplatin, whereas the overexpression of *hsa‐miR‐22‐3p* and *hsa‐miR‐200a‐3p* was linked to resistance against testosterone and bortezomib (*p* < 0.0001, logFC ≥ 2 or ≤ −2). This study provides in silico evidence for the role of dietary miRNAs in chemotherapy response, paving the way for their translational application in nutrition‐based cancer management strategies. Further experimental studies are required to quantify their bioavailability post‐digestion and to characterize their cellular uptake mechanisms.

## Graphical abstract

Dietary microRNAs (Xeno‐miRs), especially those homologous to human miRNAs found in animal‐based foods, may influence chemotherapy responses across cancer types. This study highlights the underexplored potential of human homolog egg‐derived Xeno‐miRs as biomarkers and therapeutic targets. The downregulation of hsa‐miR‐30a‐5p and hsa‐miR‐146a‐5p was linked to increased sensitivity to fluorouracil and oxaliplatin, while overexpression of hsa‐miR‐22‐3p and hsa‐miR‐200a‐3p correlated with resistance to testosterone and bortezomib, respectively (*p* < 0.0001, logFC ≥ 2 or ≤ −2). By leveraging in silico analyses, the research uncovers novel insights into the interplay between diet‐derived miRNAs and cancer treatments, paving the way for innovative nutrition‐based interventions.

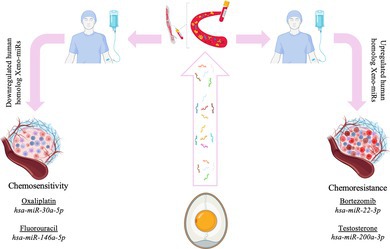

## 1 Introduction

MicroRNAs (miRNAs) are small, non‐coding RNA molecules of roughly 22 nucleotides in length (Gumusoglu‐Acar et al. 2023; Wei et al. 2024). They are involved in RNA silencing and regulate gene expression by binding to their mRNA target site(s) (Dogan et al. 2022; Wei et al. 2024). MiRNAs regulate fundamental cellular and biological functions, such as proliferation, apoptosis, and development (Sempere et al. 2021; Wu et al. 2021). Moreover, miRNAs can be detected in different body fluids and are considered promising biomarkers in various clinical conditions and environmental exposures (Chakrabortty et al. 2023; Takizawa et al. 2022).

Exogenous microRNAs (Xeno‐miRs) are a miRNA family, mostly of dietary origin, and can be detected in host biofluids (Deveci et al. 2023). Increasing evidence sheds light on the potential cross‐kingdom transfer of diet‐derived Xeno‐miRs from dietary sources into organisms, along with their ability to influence host gene expression (Guzmán‐Lorite et al. 2023; Martino et al. 2024). In the scope of this, the identification and validation of transfer Xeno‐miRs from dietary source to host organism are crucial to the underlying risk factors for developing complex diseases, such as cancer and cardiovascular diseases (Deveci et al. 2023; Guzmán‐Lorite et al. 2023; Pirim and Dogan 2020; Zhang et al. 2019).

Although one of the main food sources of humans, limited studies have identified the yolk and albumen of chicken eggs as a dietary source of miRNAs in recent decades (Fratantonio et al. 2023; Martino et al. 2024; Wade et al. 2016). The active transport of these miRNAs from the mother into the albumen or yolk is thought to help promote normal chick development (Wade et al. 2016). However, the potential transfer of chicken egg‐derived miRNAs to other species, as well as their stability and bioactivity in human systems, has yet to be elucidated (Fratantonio et al. 2023; Martino et al. 2024). Egg‐derived Xeno‐miRs may be absorbed through dietary intake and could modulate cancer‐related pathways.

Dysregulation of miRNAs is a hallmark of various diseases, especially cancer, by regulating tumor progression, metastasis, and response to chemotherapy (Iacomino 2023; Si et al. 2019). MiRNAs could serve as critical regulators of chemotherapy sensitivity, highlighting the importance of non‐coding RNAs in regulating drug resistance (Jing et al. 2020; Ye et al. 2018). Xeno‐miRs‐based therapeutics may either enhance drug efficacy or promote resistance mechanisms (Pirim and Dogan 2020). However, no studies have yet explored the specific contribution of dietary Xeno‐miRs to chemotherapy response. In this context, in silico analyses examine the potential interactions between Xeno‐miRs and chemotherapy resistance networks, offering new insights into their functional roles.

This study aims to uncover new perspectives of knowledge of egg‐derived miRNAs in cancer therapy management through in silico approaches. Egg‐derived miRNAs may influence chemotherapy outcomes and pinpoint candidate therapeutic targets, thereby generating new insights into the cross‐species regulatory potential of egg‐derived miRNAs in cancer biology.

## 2 Materials and Methods

### 2.1 Homology Analysis of Dietary Xeno‐miRs From Chicken Eggs

**FIGURE 1:**
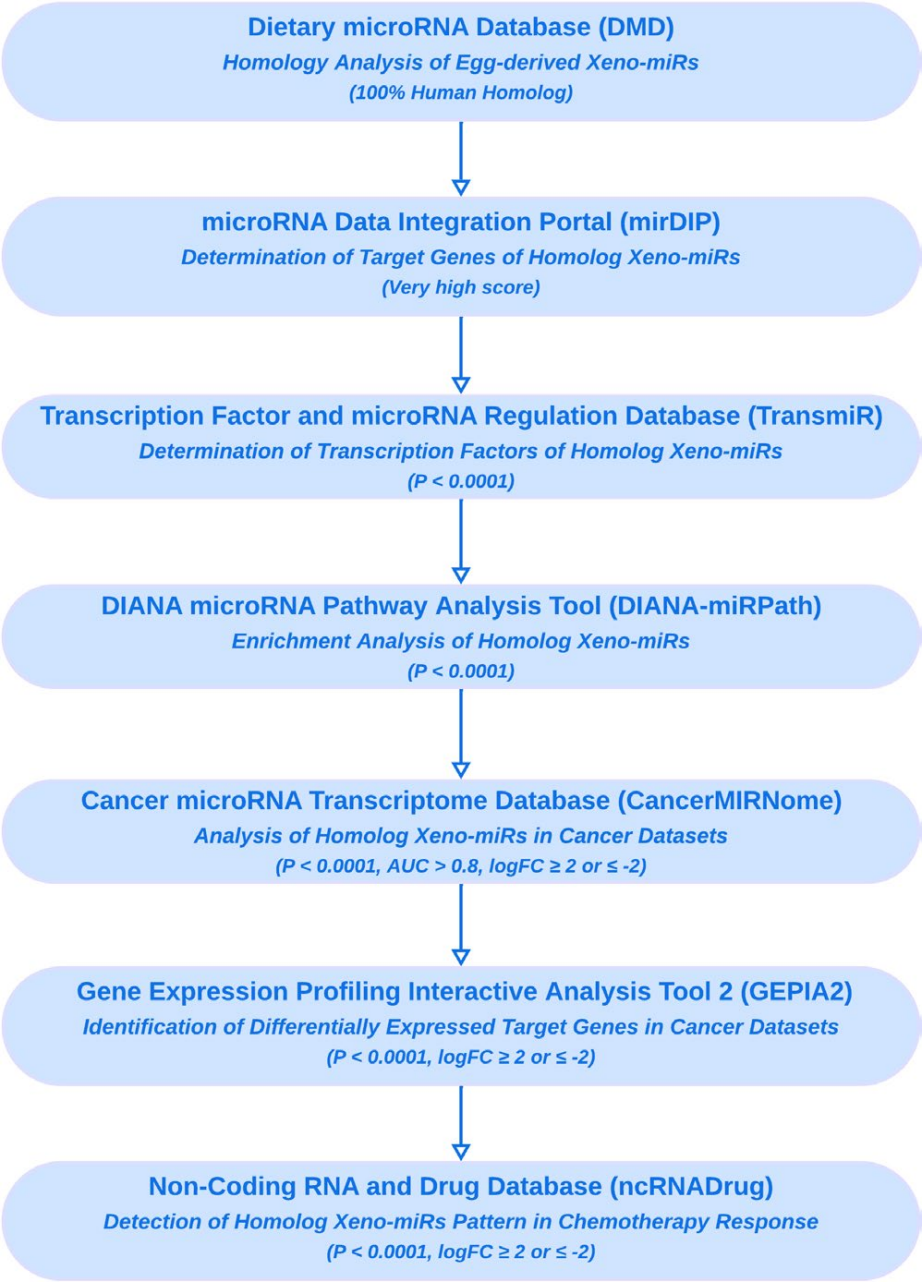
The figure depicts the workflow of the study and uses bioinformatics tools and key parameters.

In this study, 55 egg miRNAs, previously identified in chicken egg yolk through small RNA sequencing analysis, were included in the computational analysis (Fratantonio et al. 2023). To assess the cross‐species sequence comparison of miRNAs, the Dietary microRNA Database (DMD) conducted sequence alignment and comparison using the Cluster Database at High Identity with Tolerance (CD‐HIT) algorithm (Fu et al. 2012). This analysis identified homologs of the 55 egg‐derived Xeno‐miRs in *Homo sapiens* that were 100% similar to the human miRNAs, and they were included in further downstream analyses (Figure 1). The DMD integrates 5217 dietary miRNAs across 16 species, providing sequence/structure data, experimental and predicted targets (including human), functional pathways, and gene interaction annotations from public databases (Chiang et al. 2015).

### 2.2 Target Gene and Transcription Factor Prediction of Homolog Xeno‐miRs

The overlapping target genes of all human homologs of Xeno‐miRs were comprehensively analyzed using the microRNA Data Integration Portal (mirDIP) (v.5.2.3.1) database, which aggregates data from miRTarBase, miRDB, TargetScan, and DIANA‐microT databases. The database assigns confidence scores to miRNA–target interaction through a noisy‐OR model, aggregating evidence from all source datasets. These scores classify interactions into four categories labeled “very high”, “high”, “medium” and “low” confidence, corresponding to ranks (Tokar et al. 2018). The “very high” score class was applied for target gene prediction of Xeno‐miR groups in the mirDIP database.

Experimentally validated transcription factors (TFs) that bind to miRNA promoters were assessed using the Transcription Factor and microRNA Regulation (TransmiR) database (v.2.0), which provides regulatory relations between TFs and miRNAs. The TransmiR database retrieves data from literature‐curated sources and collects tissue‐specific TF binding motifs from chromatin immunoprecipitation sequencing (ChIP‐Seq) studies. TransmiR predicts TF‐miRNA regulations by scanning miRNA promoter regions (2 kb upstream of transcription start sites) against conserved TF binding motifs from the UCSC Genome Browser (hg38 assembly) and the Joint Academic Sequence Prediction and Regulation (JASPAR) 2022 database (Tong et al. 2019). Fisher's Exact test (hypergeometric test) was used to determine TF‐miRNA interaction at the set level (2). *p* values for all miRNA sets were adjusted using both Bonferroni and False Discovery Rate (FDR) corrections, with significance defined as *p* < 0.0001.

### 2.3 Pathway and Functional Enrichment Analysis of Homolog Xeno‐miRs

To evaluate the functions of homolog Xeno‐miRs, significant pathway analysis was performed using the DIANA microRNA Pathway Analysis Tool (DIANA‐miRPath) (v.4.0). DIANA‐miRPath integrates predicted and experimentally validated miRNA datasets from multiple databases, including the Kyoto Encyclopedia of Genes and Genomes (KEGG) pathways, Gene Ontology (GO) consortium, and Reactome Pathway Database (Tastsoglou et al. 2023). KEGG pathway data were used for pathway analysis, with publication permission obtained per KEGG's terms of use (Kanehisa and Goto 2000). To analyze homolog Xeno‐miRs, only “strong” targets from miRTarBase—experimentally validated targets—were selected, and the “genes union” merging method was applied. Enrichment of miRNA‐targeted genes in KEGG pathways and GO term analyses using Fisher's Exact test with FDR (Benjamini‐Hochberg). The statistical significance threshold was set at *p* < 0.0001 (FDR‐adjusted).

### 2.4 Expression Profiling of Homolog Xeno‐miRs in Cancer Datasets

The expression profiles and Receiver Operating Characteristic (ROC) analysis of homolog Xeno‐miRs were assessed using the Cancer microRNA Transcriptome (CancerMIRNome) database, which integrates miRNA expression data from The Cancer Genome Atlas (TCGA) and circulating miRNome datasets. Differential expression analysis of TCGA miRNA data was performed using the moderate *t*‐test‐based approach with an FDR < 0.05 threshold (Li et al. 2022). Xeno‐miRNA expression levels were compared between primary tumors and matched normal tissues across 33 cancer datasets. The statistical significance threshold for all analyses was set at FDR‐adjusted *p* < 0.0001, with a logarithmic fold change (logFC) ≥ 2 or ≤ −2 and an area under the curve (AUC) > 0.8. These stringent criteria were applied to enhance the robustness of the findings and mitigate potential biases arising from tumor‐normal sample size imbalance in TCGA datasets.

### 2.5 Differential Expression Analysis of Target Genes in Cancer Datasets

All target genes of human homologs of Xeno‐miR were examined in RNA‐seq data from TCGA and the Genotype‐Tissue Expression (GTEx) projects using GEPIA2 (Gene Expression Profiling Interactive Analysis 2) (Tang et al. 2019). Differentially expressed target genes were analyzed separately within RNA sequencing data of each cancer dataset using the Wilcoxon rank‐sum test. Genes with *p* < 0.0001 (FDR‐adjusted) and logFC ≥ 2 or ≤ −2 were identified as significantly differentially expressed.

### 2.6 Chemotherapy Response Patterns of Homolog Xeno‐miRs

The non‐coding RNA and drug database (ncRNADrug) was used to predict transcriptomic signatures related to drug response and target. This database provides a comprehensive search capability from the published papers, Gene Expression Omnibus (GEO) database, and extracted information from NCI‐60, TANRIC, Genomics of Drug Sensitivity in Cancer (GDSC), and Cancer Cell Line Encyclopedia (CCLE) projects. miRNA‐drug resistance pairs are initially identified by integrating multiple statistical approaches using Fisher's Exact Test [FDR < 0.01, Odds Ratio (OR) > 2] for binary resistance classification, followed by Spearman correlation (Cao et al. 2024). The Xeno‐miRs pattern of chemotherapy response was identified in the GEO repository with criteria of logFC ≥ 2 or ≤ −2 and *p* < 0.0001. Xeno‐miRs showing significant dual effects (both resistance and sensitivity) were excluded.

## 3 Results

### 3.1 Human Homologs of Egg‐Derived Xeno‐miRs

Human homologs of 55 transferrable egg‐derived Xeno‐miRs candidates from *Gallus gallus* were analyzed from DMD. The characteristics of the 55 Xeno‐miRs identified in this study, including their sequence, length, miRBase IDs, and homologs in humans and other species, are provided in Table S1. Xeno‐miRs showing 100% homology to human miRNAs were included for further analyses. Of these, 37 Xeno‐miRNAs did not have any human homologs. Three Xeno‐miRs, *gga‐miR‐23b‐3p*, *gga‐miR‐30d*, and *gga‐miR‐200a‐3p*, were found to have homologs only in humans. Other Xeno‐miRs had homologs in both humans and the various dietary species. Additionally, 12 Xeno‐miRs (*gga‐miR‐26a‐5p*, *gga‐miR‐30e‐3p*, *gga‐miR‐30e‐5p*, *gga‐miR‐92‐3p*, *gga‐miR‐103‐3p*, *gga‐miR‐125b‐3p*, *gga‐miR‐126‐3p*, *gga‐miR‐133c‐3p*, *gga‐miR‐140‐3p*, *gga‐miR‐142‐5p*, *gga‐miR‐181a‐3p*, *gga‐miR‐221‐3p*) were specific to *G. gallus* , with no homologous dietary species found in the database. The sequences of *gga‐let‐7a‐5p* and *gga‐let‐7j‐5p*, as well as *gga‐miR‐204* and *gga‐miR‐211*, were identical but named differently in the database; thus, these Xeno‐miRNAs were considered the same. Our homology analysis revealed 18 transportable Xeno‐miRs from chicken eggs that are 100% similar to the human miRNAs. Table 1 lists the annotations of these 18 egg‐derived Xeno‐miRs along with their 17 human homologs and species‐specific miRBase IDs.

**TABLE 1:** Annotations of human homologs of 18 egg‐derived Xeno‐miRs showing 100% homology.

Xeno‐miR	miRBase ID ( *Gallus gallus* )	Sequence	Length	Human homolog	miRBase ID ( *Homo sapiens* )
*gga‐let‐7a‐5p* ^a^	MIMAT0001101	UGAGGUAGUAGGUUGUAUAGUU	22	*hsa‐let‐7a‐5p*	MIMAT0000062
*gga‐let‐7j‐5p* ^a^	MIMAT0001181	UGAGGUAGUAGGUUGUAUAGUU	22	*hsa‐let‐7a‐5p*	MIMAT0000062
*gga‐miR‐9‐5p*	MIMAT0001195	UCUUUGGUUAUCUAGCUGUAUGA	23	*hsa‐miR‐9‐5p*	MIMAT0000441
*gga‐miR‐21‐5p*	MIMAT0003774	UAGCUUAUCAGACUGAUGUUGA	22	*hsa‐miR‐21‐5p*	MIMAT0000076
*gga‐miR‐22‐3p*	MIMAT0007288	AAGCUGCCAGUUGAAGAACUGU	22	*hsa‐miR‐22‐3p*	MIMAT0000077
*gga‐miR‐23b‐3p*	MIMAT0001186	AUCACAUUGCCAGGGAUUACC	21	*hsa‐miR‐23b‐3p*	MIMAT0000418
*gga‐miR‐27b‐3p*	MIMAT0001187	UUCACAGUGGCUAAGUUCUGC	21	*hsa‐miR‐27b‐3p*	MIMAT0000419
*gga‐miR‐30a‐5p*	MIMAT0001135	UGUAAACAUCCUCGACUGGAAG	22	*hsa‐miR‐30a‐5p*	MIMAT0000087
*gga‐miR‐30d*	MIMAT0001129	UGUAAACAUCCCCGACUGGAAG	22	*hsa‐miR‐30d‐5p*	MIMAT0000245
*gga‐miR‐99a‐5p*	MIMAT0001103	AACCCGUAGAUCCGAUCUUGUG	22	*hsa‐miR‐99a‐5p*	MIMAT0000097
*gga‐miR‐103‐3p*	MIMAT0001145	AGCAGCAUUGUACAGGGCUAUGA	23	*hsa‐miR‐103a‐3p*	MIMAT0000101
*gga‐miR‐107‐3p*	MIMAT0001147	AGCAGCAUUGUACAGGGCUAUCA	23	*hsa‐miR‐107*	MIMAT0000104
*gga‐miR‐130c‐3p*	MIMAT0007734	CAGUGCAAUGUUAAAAGGGCAU	22	*hsa‐miR‐130a‐3p*	MIMAT0000425
*gga‐miR‐146a‐5p*	MIMAT0001163	UGAGAACUGAAUUCCAUGGGUU	22	*hsa‐miR‐146a‐5p*	MIMAT0000449
*gga‐miR‐148a‐3p*	MIMAT0001120	UCAGUGCACUACAGAACUUUGU	22	*hsa‐miR‐148a‐3p*	MIMAT0000243
*gga‐miR‐181a‐5p*	MIMAT0001168	AACAUUCAACGCUGUCGGUGAGU	23	*hsa‐miR‐181a‐5p*	MIMAT0000256
*gga‐miR‐200a‐3p*	MIMAT0001171	UAACACUGUCUGGUAACGAUGU	22	*hsa‐miR‐200a‐3p*	MIMAT0000682
*gga‐miR‐205a*	MIMAT0001184	UCCUUCAUUCCACCGGAGUCUG	22	*hsa‐miR‐205‐5p*	MIMAT0000266

Abbreviations: *gga, Gallus gallus*; *hsa, Homo sapiens*.

^a^ Xeno‐miRs with the same sequences.

### 3.2 Target Genes and Transcription Factor Analyses of Homolog of Xeno‐miRs

**FIGURE 2:**
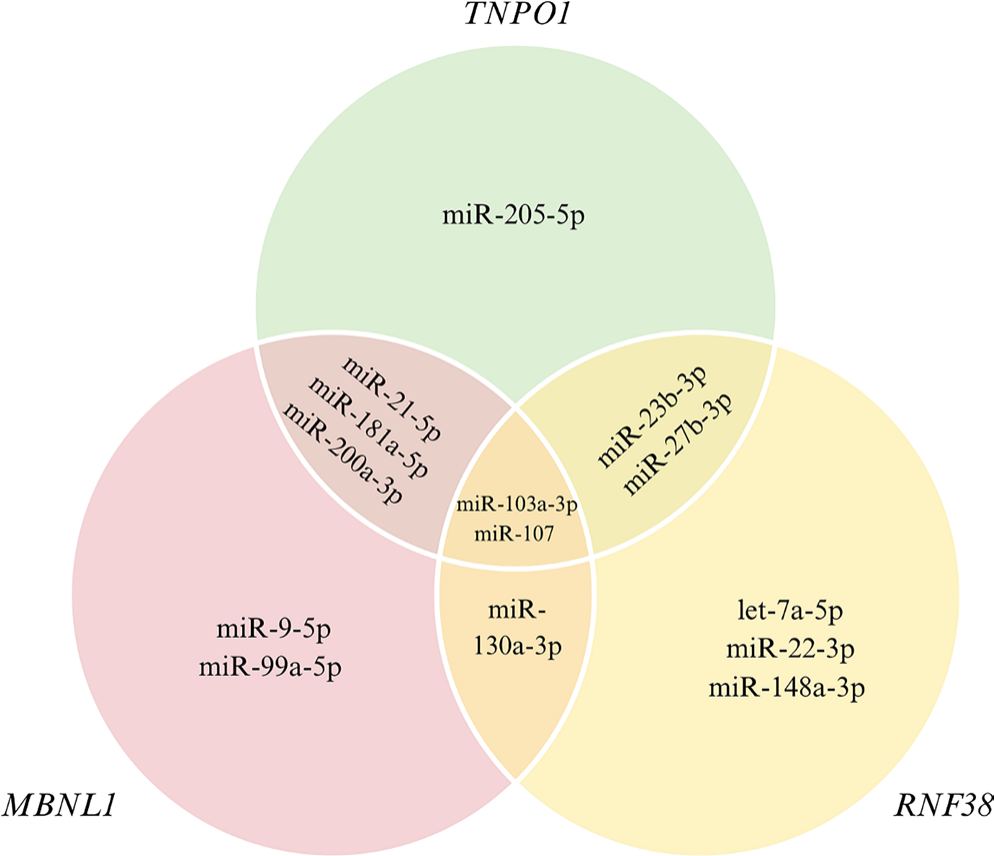
Venn diagram showing the target genes identified for homolog Xeno‐miRs, highlighting the shared and overlapping target genes among egg‐derived homolog Xeno‐miRs.

The potential target genes of the 17 homolog Xeno‐miRs were predicted using the mirDIP database. The target genes were determined based on a “very high” filter and were targeted by at least 8 homolog Xeno‐miRs. Figure 2 illustrates the overlapping target genes of Xeno‐miRs, potentially co‐regulated by multiple Xeno‐miRs. The *hsa‐miR‐103a‐3p* and *hsa‐miR‐107* were found to target all the identified target genes. Additionally, the Muscleblind Like Splicing Regulator 1 (*MBNL1*), Ring Finger Protein 38 (*RNF38*) and Transportin 1 (*TNPO1*) genes were identified as targets of the highest number of homolog Xeno‐miRs.

**FIGURE 3:**
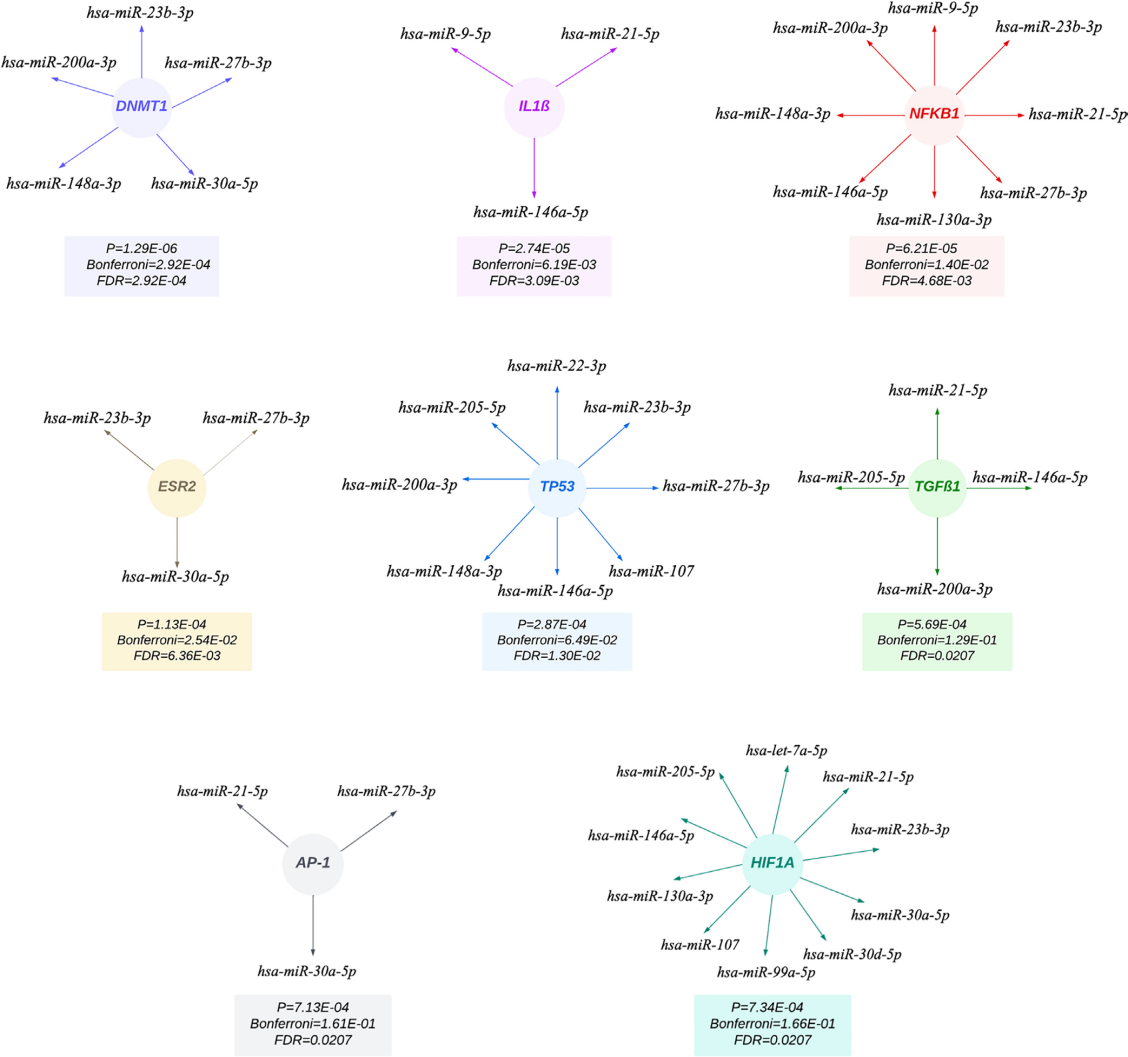
Identification of TFs of homolog Xeno‐miRs with enrichment analysis (*p* < 0.0001). AP‐1, Activating Protein‐1; DNMT1, DNA Methyltransferase 1; ESR2, Estrogen Receptor 2; FDR, False Discovery Rate; HIF1A, Hypoxia Inducible Factor 1 Subunit Alpha; IL1ß, Interleukin 1 Beta; NFKB1, Nuclear Factor Kappa B Subunit 1; TGFß1, Transforming Growth Factor Beta 1; TFs, Transcription Factors; TP53, Tumor Protein P53.

The enrichment analysis of TF‐miRNA interactions revealed significant regulatory relationships between 15 Xeno‐miRs and 8 key TFs (*p* < 0.0001) (Figure 3). Each TF of homolog Xeno‐miRs is visualized in Figure 3, along with *p* value, Bonferroni, and FDR values. These interactions revealed DNA Methyltransferase 1 (*DNMT1*) as the most significant transcription factor (*p* = 1.29E‐06). No interactions were observed between the TFs and 2 specific miRNAs (*hsa‐miR‐181a‐5p* and *hsa‐miR‐200a‐3p*).

### 3.3 KEGG Pathway and GO Terms Analyses of Homolog Xeno‐miRs

**FIGURE 4:**
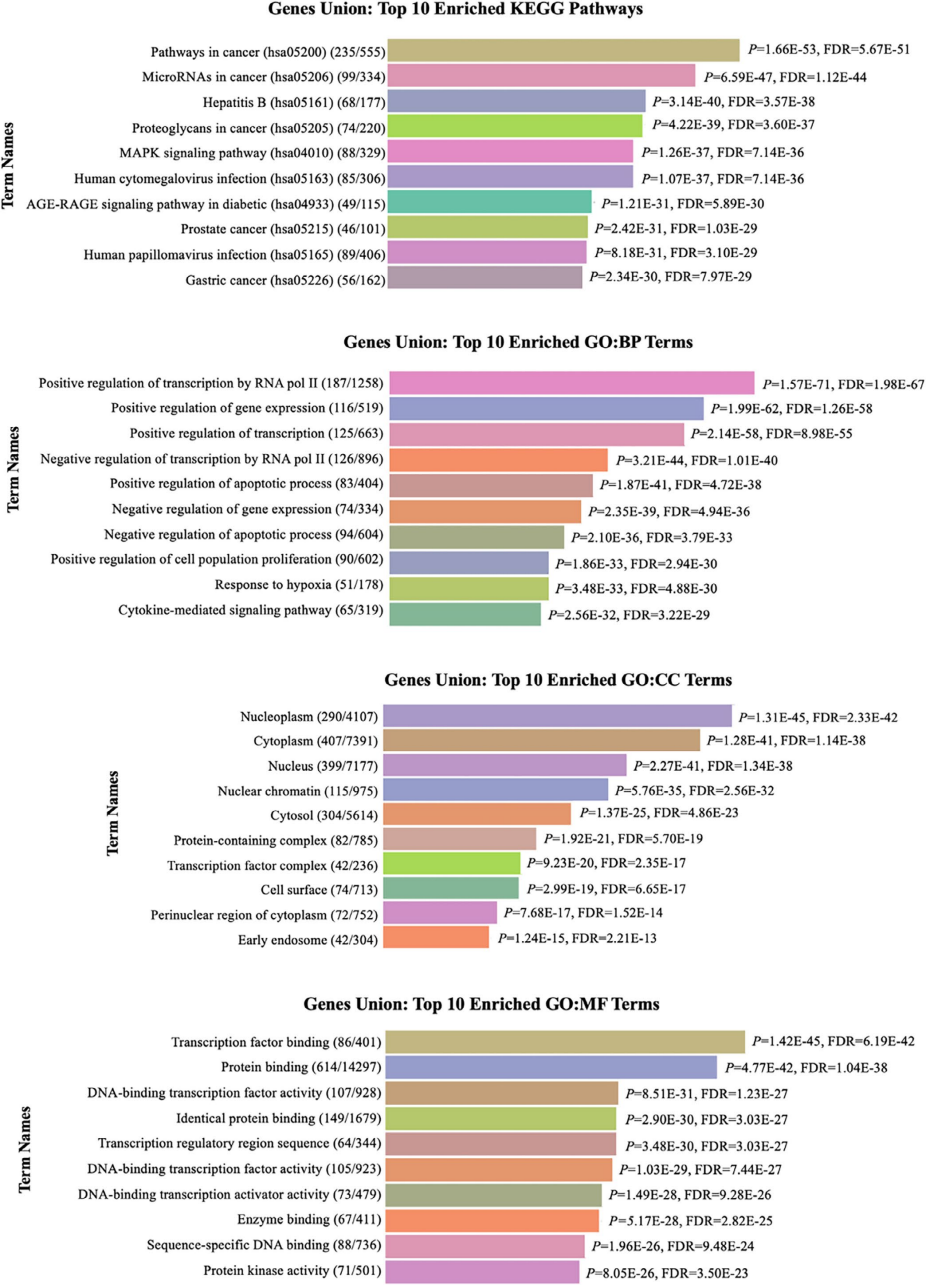
The top ten significant KEGG pathways and GO terms were identified by enrichment analyses of human homologs of 17 Xeno‐miRs (*p* < 0.0001).

The enrichment pathway analysis of miRNAs was conducted using DIANA‐miRPath (v.4.0), focusing on KEGG pathways and Gene Ontology (GO) terms. The “Pathways in Cancer” (KEGG ID: hsa05206) emerged as the most significant pathway. The top ten significantly enriched KEGG pathways and GO terms, including Biological Process (BP), Cellular Component (CC), and Molecular Function (MF), associated with the human homologs of 17 Xeno‐miRs (*p* < 0.0001) are provided in Figure 4. These results provide insights into the potential regulatory roles of Xeno‐miRs in cancer‐related pathways and cellular functions.

### 3.4 Expression Patterns of Homolog Xeno‐miRs in Cancer Datasets

The expression patterns of the homolog Xeno‐miRs were investigated using TCGA cancer datasets in the CancerMIRNome database. Among these, hsa‐miR‐21‐5p and hsa‐miR‐200a‐3p were most associated with cancer types significant in 12 different cancer types. Colorectal Adenocarcinoma (COAD) was associated with the highest number of significant homolog Xeno‐miRs, while Kidney Renal Papillary Cell Carcinoma (KIRP) and Thyroid Carcinoma (THCA) had the least interaction. No significant interaction was observed in cancer datasets with *hsa‐let‐7a‐5p*, *hsa‐miR‐103a‐3p*, and *hsa‐miR‐107*. The logFC, *p* value, adjusted *p* value, AUC values, and *p* value of ROC results for homolog Xeno‐miRs in cancer datasets are detailed in Table 2.

**TABLE 2:** Differential expression of homolog Xeno‐miRs across TCGA datasets (*p* < 0.0001, AUC > 0.8, and logFC ≥ 2 or ≤ −2).

		*hsa‐miR‐9‐5p*	*hsa‐miR‐21‐5p*	*hsa‐miR‐22‐3p*	*hsa‐miR‐23b‐3p*	*hsa‐miR‐27b‐3p*	*hsa‐miR‐30a‐5p*	*hsa‐miR‐30d‐5p*	*hsa‐miR‐99a‐5p*	*hsa‐miR‐130a‐3p*	*hsa‐miR‐146a‐5p*	*hsa‐miR‐148a‐3p*	*hsa‐miR‐181a‐5p*	*hsa‐miR‐200a‐3p*	*hsa‐miR‐205‐5p*
TCGA‐BLCA N (*n* = 19) T (*n* = 428)	logFC						−2.11							3.39	2.91
	*p*						1.13E‐14							4.76E‐12	2.34E‐07
	Adj. *p*						1.18E‐13							3.37E‐11	7.76E‐07
	AUC						0.94							0.85	0.81
	ROC *p*						6.10E‐103							9.76E‐65	3.64E‐50
TCGA‐BRCA N (*n* = 104) T (*n* = 1182)	logFC		2.29						−2.36					2.69	
	*p*		8.90E‐127						1.28E‐66					1.27E‐72	
	Adj. *p*		2.05E‐124						2.57E‐65					3.67E‐71	
	AUC		0.97						0.95					0.92	
	ROC *p*		1.39E‐231						8.18E‐239					6.36E‐204	
TCGA‐CHOL N (*n* = 9) T (*n* = 45)	logFC		2.47						−2.44			−2.89		2.51	
	*p*		5.14E‐12						8.40E‐10			1.49E‐14		3.76E‐07	
	Adj. *p*		2.54E‐10						1.70E‐08			1.65E‐12		3.21E‐06	
	AUC		1						1			1		0.97	
	ROC *p*		8.31E‐12						1.32E‐11			5.54E‐12		1.38E‐10	
TCGA‐COAD N (*n* = 8) T (*n* = 452)	logFC		7.07	2.19						2.06	2.26	4.09	−2.91	4.09	
	*p*		1.41E‐101	3.90E‐18						9.12E‐12	7.07E‐09	3.34E‐37	7.66E‐20	3.06E‐32	
	Adj. *p*		6.57E‐99	1.37E‐17						2.23E‐11	1.44E‐08	2.16E‐36	2.88E‐19	1.68E‐31	
	AUC		1	0.99						0.97	0.93	1	0.99	1	
	ROC *p*		7.82E‐147	1.01E‐141						2.27E‐125	4.29E‐105	1.12E‐151	3.78E‐139	1.49E‐148	
TCGA‐HNSC N (*n* = 44) T (*n* = 567)	logFC						−2.01		−2.57						
	*p*						1.75E‐34		9.03E‐27						
	Adj. *p*						8.50E‐33		1.37E‐25						
	AUC						0.94		0.95						
	ROC *p*						8.15E‐121		5.94E‐125						
TCGA‐KICH N (*n* = 25) T (*n* = 91)	logFC	−2.37								−2.49					
	*p*	3.39E‐05								9.13E‐25					
	Adj. *p*	7.65E‐05								4.64E‐23					
	AUC	0.82								1					
	ROC *p*	7.03E‐10								1.94E‐20					
TCGA‐KIRC N (*n* = 71) T (*n* = 587)	logFC	−2.57	2.39												
	*p*	7.08E‐18	1.74E‐82												
	Adj. *p*	2.48E‐17	1.86E‐80												
	AUC	0.83	9.60E‐01												
	ROC *p*	1.83E‐70	2.50E‐121												
TCGA‐KIRP N (*n* = 34) T (*n* = 325)	logFC		3.38												
	*p*		1.68E‐66												
	Adj. *p*		6.72E‐64												
	AUC		9.80E‐01												
	ROC *p*		6.48E‐81												
TCGA‐LUAD N (*n* = 46) T (*n* = 559)	logFC	4.21	2.73											2.27	
	*p*	1.58E‐29	4.58E‐51											6.57E‐24	
	Adj. *p*	1.88E‐28	3.04E‐49											5.08E‐23	
	AUC	0.95	0.94											0.88	
	ROC *p*	2.90E‐125	6.14E‐116											1.39E‐92	
TCGA‐LUSC N (*n* = 45) T (*n* = 478)	logFC	4.47					−3.39	−2.67							6.65
	*p*	1.66E‐42					2.82E‐72	1.70E‐78							1.80E‐82
	Adj. *p*	2.67E‐41					3.51E‐70	2.82E‐76							4.49E‐80
	AUC	0.97					0.98	0.97							0.97
	ROC *p*	5.77E‐128					1.24E‐128	1.50E‐123							9.48E‐128
TCGA‐THCA N (*n* = 59) T (*n* = 506)	logFC	−2.15													
	*p*	1.34E‐28													
	Adj. *p*	3.66E‐27													
	AUC	0.9													
	ROC *p*	2.49E‐98													
TCGA‐UCEC N (*n* = 33) T (*n* = 538)	logFC				−2.23	−2.45			−3.91					3.66	4.91
	*p*				3.36E‐43	2.91E‐45			9.68E‐40					2.61E‐44	1.47E‐24
	Adj. *p*				8.05E‐42	8.14E‐44			1.80E‐38					6.90E‐43	1.06E‐23
	AUC				0.97	0.99			0.99					0.94	0.93
	ROC *p*				6.68E‐142	6.94E‐157			3.73E‐166					4.71E‐119	1.07E‐124

Abbreviations: adj. *p*, Adjusted *p* Value; BLCA, Bladder Carcinoma; BRCA, Breast Invasive Carcinoma; CHOL, Cholangiocarcinoma; COAD, Colon Adenocarcinoma; HNSC, Head and Neck Squamous Cell Carcinoma; KICH, Kidney Chromophobe; KIRC, Kidney Renal Clear Cell Carcinoma; KIRP, Kidney Renal Papillary Cell Carcinoma; logFC, Logarithmic Fold Change; LUAD, Lung Adenocarcinoma; LUSC, Lung Squamous Cell Carcinoma; N, Normal samples; T, Tumor samples; THCA, Thyroid Carcinoma; UCEC, Uterine Corpus Endometrial Carcinoma.

### 3.5 Differentially Expressed Target Genes of Homolog Xeno‐miRs Using TCGA Datasets

**FIGURE 5:**
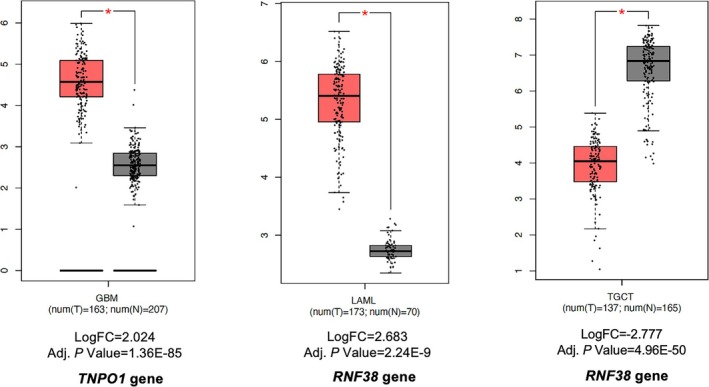
Expression profiles of target genes of homolog Xeno‐miRs in cancer datasets. Orange and gray represent tumor and normal samples, respectively (*p* < 0.0001 and logFC ≥ 2 or ≤ −2). adj. *p*, Adjusted *p* Value; GBM, Glioblastoma Multiforme; LAML, Acute Myeloid Leukemia; logFC, Logarithmic fold change; N, Normal samples; T, Tumor samples; TGCT, Testicular Germ Cell Tumors.

Differential gene expression analysis was performed to assess the expressions of identified genes in the TCGA cancer datasets utilizing the GEPIA2 database. Three interactions involving two differentially expressed genes (*p* < 0.0001) were identified across three cancer types when comparing cancer tissues with paired normal tissues. Box plots of differentially expressed target genes in different cancer types and their logFC and adjusted *p* values are provided in Figure 5.

### 3.6 Impact of Homolog Xeno‐miRs on Chemotherapy Response

The relationship between Xeno‐miRs expression profiles and chemotherapy response was further analyzed. Twenty‐three Xeno‐miRs interactions involving 12 chemotherapy agents were identified (logFC ≥ 2 or ≤ −2 and *p* < 0.0001). Table 3 presents the expression pattern, logFC, and *p* value of homolog Xeno‐miRs in response to chemotherapy agents. Among these interactions, 10 were identified as resistant, while 13 were linked to sensitivity. Fluorouracil and oxaliplatin exhibited the highest relationships with homolog Xeno‐miRs (*n* = 4). Among the homolog Xeno‐miRs, *hsa‐miR‐181a‐5p* was most commonly associated with chemotherapy agents (*n* = 6). Of the 17 homologs Xeno‐miRs analyzed, 7 Xeno‐miRs (*hsa‐let‐7a‐5p*, *hsa‐miR‐21‐5p*, *hsa‐miR‐30d‐5p*, *hsa‐miR‐103a‐3p*, *hsa‐miR‐107*, *hsa‐miR‐130a‐3p* and *hsa‐miR‐148a‐3p*) showed no interactions with any chemotherapy agents. In 10 Xeno‐miRs with observed interactions, 6 Xeno‐miRs showed dual patterns across different chemotherapy agents. The results indicate that downregulated miRNAs are associated with drug sensitivity patterns, while upregulated miRNAs are linked to drug resistance patterns. The h*sa‐miR‐30a‐5p* and *hsa‐miR‐146a‐5p* were identified as sensitivity‐specific, whereas *hsa‐miR‐200a‐3p* and *hsa‐miR‐22‐3p* were associated exclusively with resistance across different chemotherapy agents. Especially, *hsa‐miR‐205‐5p*, *hsa‐miR‐23b‐3p*, and *hsa‐miR‐27b‐3p* for oxaliplatin and *hsa‐miR‐181a‐5p*, *hsa‐miR‐9‐5p*, and *hsa‐miR‐99a‐5p* for fluorouracil were found in the resistant pattern. Moreover, *hsa‐miR‐181a‐5p*, *hsa‐miR‐205‐5p*, and *hsa‐miR‐99a‐5p* for testosterone+exemestane were found in a sensitive pattern.

**TABLE 3:** Expression profile of homolog Xeno‐miRs in response to chemotherapy agents logFC ≥ 2 or ≤ −2 and *p* < 0.0001.

Chemotherapy agents	miRNA	Pattern	logFC	*p*
*Down regulation*				
Vemurafenib	*hsa‐miR‐27b‐3p*	Sensitive	−2.535	1.92E‐42
Vemurafenib	*hsa‐miR‐23b‐3p*	Sensitive	−2.802	3.95E‐42
Fluorouracil	*hsa‐miR‐146a‐5p*	Sensitive	−3.973	8.23E‐40
Oxaliplatin	*hsa‐miR‐30a‐5p*	Sensitive	−5.252	1.02E‐23
Exemestane	*hsa‐miR‐181a‐5p*	Sensitive	−2.682	3.18E‐08
Testosterone + Exemestane	*hsa‐miR‐181a‐5p*	Sensitive	−2.566	6.12E‐08
Testosterone + Exemestane	*hsa‐miR‐99a‐5p*	Sensitive	−2.118	1.29E‐07
Testosterone + Letrozole	*hsa‐miR‐181a‐5p*	Sensitive	−2.421	1.42E‐07
Testosterone + Anastrozole	*hsa‐miR‐181a‐5p*	Sensitive	−2.327	2.49E‐07
Temozolomide	*hsa‐miR‐9‐5p*	Sensitive	−2.078	5.58E‐07
Testosterone	*hsa‐miR‐181a‐5p*	Sensitive	−2.159	3.10E‐06
Exemestane	*hsa‐miR‐205‐5p*	Sensitive	−2.461	3.66E‐05
Testosterone + Exemestane	*hsa‐miR‐205‐5p*	Sensitive	−2.421	4.41E‐05
*Up regulation*				
Bortezomib	*hsa‐miR‐22‐3p*	Resistant	7.402	3.32E‐18
Fluorouracil	*hsa‐miR‐9‐5p*	Resistant	2.491	4.35E‐15
Oxaliplatin	*hsa‐miR‐23b‐3p*	Resistant	2.457	6.08E‐14
Oxaliplatin	*hsa‐miR‐27b‐3p*	Resistant	2.155	8.04E‐13
Fluorouracil	*hsa‐miR‐181a‐5p*	Resistant	2.106	6.33E‐10
Testosterone	*hsa‐miR‐200a‐3p*	Resistant	2.08	5.80E‐09
Fluorouracil	*hsa‐miR‐99a‐5p*	Resistant	2.275	4.86E‐08
Testosterone + Tamoxifen	*hsa‐miR‐27b‐3p*	Resistant	2.084	2.91E‐07
Oxaliplatin	*hsa‐miR‐205‐5p*	Resistant	5.802	1.34E‐06
Ceritinib	*hsa‐miR‐205‐5p*	Resistant	6.042	9.95E‐05

## 4 Discussion

The growing knowledge of various aspects of carcinogenesis and their implications for therapeutic applications suggests that miRNAs are promising candidates for predicting treatment responses (Lang et al. 2022; Tsotridou et al. 2024; Ueda et al. 2024). However, the therapeutic potential of dietary Xeno‐miRs and their influence on chemotherapy response has yet to be fully investigated. This is the first study to propose a potential link between dietary miRNAs and chemotherapy outcomes. Our findings provide evidence that egg‐derived human homolog egg‐derived Xeno‐miRs may significantly influence chemotherapy response (Table 3). These results shed important light on the possible functions of egg‐derived Xeno‐miRs in human cancer management, particularly in relation to chemotherapy sensitivity and resistance.

Growing evidence suggests that dietary miRNAs from animal sources may exert cross‐species regulatory effects in humans, potentially influencing critical cancer‐related pathways, as demonstrated in cancer cell cultures. Specifically, egg‐derived *miR‐181a* and *miR‐181b* regulate the expression of *BCL2* (B‐cell lymphoma 2) and *BCL2A1* (BCL2 Related Protein A1) in human plasma (Baier 2015). Marzano et al. showed that *mtr‐miR‐5754* and *gma‐miR‐4995* target and destabilize oncogenic lncRNAs Metastasis Associated Lung Adenocarcinoma Transcript 1 (*MALAT1*) and Nuclear Paraspeckle Assembly Transcript 1 (*NEAT1*), leading to reduced proliferation in HCT116 colorectal cancer cells (Marzano et al. 2020). Similarly, plant‐derived *MIR167e‐5p* suppressed β‐catenin expression in IPEC‐J2 and Caco‐2 cell lines, suggesting its role in cross‐kingdom epigenetic regulation (Li et al. 2019). Minutolo et al. revealed that natural oeu‐sRNAs downregulated the protein expression of *hsa‐miR‐34a* targets, resulting in reduced proliferation and increased apoptosis in various tumor cells (Minutolo et al. 2018). Additionally, milk‐derived *miR‐29b* and *miR‐200c* were absorbed in humans and suppressed their target genes, RUNX Family Transcription Factor 2 (*RUNX2*), in HEK‐293 cells, highlighting their bioactive role in dietary miRNA uptake (Baier et al. 2014). Our previous study has also highlighted the potential role of Xeno‐miRs from various animal species, including cows, pigs, and chickens, in regulating carcinogenesis (Pirim and Dogan 2020). These findings suggest that modulating specific dietary miRNA intake could represent a novel therapeutic approach for targeting cancer‐related pathways and enhancing chemotherapy response.

Our results demonstrated that *hsa‐miR‐30a‐5p* and *hsa‐miR‐146a‐5p* are associated with chemotherapy sensitivity, and their downregulation enhances the sensitivity of cancer cells to fluorouracil and oxaliplatin. Similarly, inhibition of the expression of *hsa‐miR‐30a* may chemosensitize oral squamous cell carcinoma cells to 5‐Fluorouracil (5‐FU) by reducing the number of cells arrested in the G1 phase and increasing cell proliferation (Kawahara et al. 2021). Conversely, *hsa‐miR‐30a‐5p* has been shown to enhance 5‐FU sensitivity in esophageal squamous cell carcinoma by downregulating Frizzled Class Receptor 3 (*FZD3*), a transmembrane receptor involved in the Wnt signaling pathway (Yao et al. 2021). Moreover, *hsa‐miR‐146a* is upregulated in 5‐FU‐resistant oesophageal cancer (Mahawongkajit and Tomtitchong 2020); however, it enhances oxaliplatin sensitivity by inhibiting aerobic glycolysis and promoting senescence in hepatocellular carcinoma cells (Yang et al. 2024).

Moreover, the overexpression of *hsa‐miR‐22‐3p* and *hsa‐miR‐200a‐3p* activates mechanisms that contribute to resistance against testosterone and bortezomib. These two miRNAs are known to regulate key processes such as cell proliferation, apoptosis, and the epithelial‐mesenchymal transition (EMT), which are involved in cancer progression and treatment outcomes. The *hsa‐miR‐22‐3p* promotes chemoresistance by targeting Neuroepithelial Cell Transforming 1 (*NET1*) in breast cancer (Xiao et al. 2018), while it enhances chemosensitivity to cisplatin through the PTEN/PI3K/Akt pathway in gastrointestinal stromal tumor cell lines (Xu et al. 2018). On the other hand, *hsa‐miR‐200a‐3p* increases 5‐FU resistance by downregulating Dual Specificity Phosphatase 6 (*DUSP6*) in hepatocellular carcinoma (Lee et al. 2017). There is no current data on the roles of *hsa‐miR‐200a‐3p* and *hsa‐miR‐22‐3p* in mediating resistance to testosterone and bortezomib, respectively. However, evidence suggests their involvement in modulating chemotherapy responses in various other cancer types.

Our results illustrate that oxaliplatin resistance has been linked to the upregulation of *hsa‐miR‐23b‐3p*, *hsa‐miR‐27b‐3p*, and *hsa‐miR‐205‐5p*. Similarly, *hsa‐miR‐23b‐3p* expression is elevated in oxaliplatin‐resistant colorectal adenocarcinoma cell lines, while oxaliplatin‐resistant colorectal cancer cells exhibit reduced levels of *hsa‐miR‐27b‐3p* (Gasiule et al. 2019; Sun et al. 2020). The *hsa‐miR‐205‐5p* enhances the chemosensitivity of renal carcinoma cells to 5‐FU and oxaliplatin by targeting Vascular Endothelial Growth Factor A (*VEGFA*) and modulating the PI3K/Akt signaling pathway (Huang et al. 2019). Correspondingly, the evidence suggests that fluorouracil resistance has been associated with the dysregulation of *hsa‐miR‐9‐5p*, *hsa‐miR‐99a‐5p*, and *hsa‐miR‐181a‐5p*. The *hsa‐miR‐181a‐5p* inhibits Wnt/β‐catenin and Pleomorphic Adenoma Gene 1 (*PLAG1*) pathways, enhancing the sensitivity of colorectal cancer cells to 5‐FU (Li et al. 2023; Shi et al. 2018). Conversely, *hsa‐miR‐9‐5p* increases 5‐FU sensitivity in colorectal cancer by downregulating High Mobility Group AT‐Hook 2 (*HMGA2*) (Zheng et al. 2021). On the other hand, *hsa‐miR‐99a‐5p* promotes cisplatin sensitivity and induces apoptosis by suppressing Very Low‐Density Lipoprotein Receptor (*VLDLR*) expression in lung cancer (Lang et al. 2023).

Despite the growing understanding of endogenous miRNAs in chemotherapy, the influence of dietary miRNAs on chemotherapy outcomes remains unclear. These findings underscore that identified miRNAs may play a significant role in modulating chemotherapy responses. However, their effects depend on the molecular characteristics of cancer type and resistance mechanisms. In addition, certain miRNAs exhibit dual roles, influencing the efficacy of specific chemotherapeutic agents in a cancer‐type‐dependent manner.

This study has several limitations due to the in silico design but offers valuable insight into the implications of animal‐derived Xeno‐miRs on cancer treatment. Firstly, the findings are restricted to analyses of the TCGA and GEO databases, requiring validation across additional datasets and diverse populations. Secondly, our results are promising to be further investigated by “wet lab” experiments to validate the role of Xeno‐miRs and their targets in chemotherapy response. Third, we excluded egg‐specific Xeno‐miRs without human homologs due to database constraints, potentially overlooking unique biological effects. Finally, some evidence suggests dietary miRNAs may pass through the gastrointestinal tract, but their bioavailability in humans remains controversial (Alshehri 2021; Benmoussa and Provost 2019; Diez‐Sainz et al. 2024; Link et al. 2019; Mar‐Aguilar et al. 2020). Further experimental studies are required to determine whether to quantify their bioavailability post‐digestion and characterize their cellular uptake mechanisms.

In conclusion, this study reveals egg‐derived homolog Xeno‐miRs as potential modulators of chemotherapy response, highlighting two key clinical applications: (1) predictive biomarkers in liquid biopsies and (2) therapeutic targets for miRNA‐based interventions. Using in silico approaches, we identified candidate Xeno‐miRs that may influence chemotherapy outcomes and serve as potential therapeutic targets. Future experimental studies should validate their bioavailability as dietary micronutrients and assess nutritional interventions to impact chemotherapy response. These findings could ameliorate cancer management by exploring the translational applicability of targeting Xeno‐miRs in clinical settings and developing novel therapeutic strategies centered on Xeno‐miRs.

## Author Contributions

**Berkcan Doğan:** conceptualization (lead), formal analysis (lead), methodology (lead), visualization (lead), writing – original draft (lead), writing – review and editing (lead).

## Conflicts of Interest

The author declares no conflicts of interest.

## Supporting information


**Table S1.** Annotations and analysis results of 55 egg‐derived Xeno‐miRs showing 100% homology in species‐specific comparisons.

## Data Availability

This study analyzed existing public databases and web tools, described in the Materials and Methods section and illustrated in the diagram. All processed results are provided in the manuscript and supplementary file.
