# Supplementary material for: The Potential Role of Egg‐Derived Xeno‐miRs in Chemotherapy Response: An In Silico Approach
Source: Food Sci Nutr. 2025 May 23;13(6):e70332. doi: 10.1002/fsn3.70332 (PMC12121447; doi:10.1002/fsn3.70332)
Supplement: Supplementary file 1 — Table S1. Annotations and analysis results of 55 egg‐derived Xeno‐miRs showing 100% homology in species‐specific comparisons. [file FSN3-13-e70332-s001.docx]

**Supplementary Information**

**Table S1.** Annotations and analysis results of 55 egg-derived Xeno-miRs showing 100% homology in species-specific comparisons.

| **Xeno-miR** | **miRBase ID (Egg)** | **Sequence** | **Length** | **Human Homolog** | **miRBase ID (Human)** | **Homolog in Other Species** |
| --- | --- | --- | --- | --- | --- | --- |
| *gga-let-7a-5p** | MIMAT0001101 | UGAGGUAGUAGGUUGUAUAGUU | 22 | *hsa-let-7a-5p* | MIMAT0000062 | *bta-let-7a-5, ssa-let-7a-5p, ssc-let-7a* |
| *gga-let-7b* | MIMAT0001102 | UGAGGUAGUAGGUUGUGUGGUU | 22 |  |  | *bta-let-7b, ssa-let-7b-5p* |
| *gga-let-7c-5p* | MIMAT0001104 | UGAGGUAGUAGGUUGUAUGGUU | 22 |  |  | *bta-let-7c, ssa-let-7c-5p, ssc-let-7c* |
| *gga-let-7f-5p* | MIMAT0001162 | UGAGGUAGUAGAUUGUAUAGUU | 22 |  |  | *bta-let-7f, ssc-let-7f* |
| *gga-let-7g-5p* | MIMAT0001160 | UGAGGUAGUAGUUUGUACAGU | 21 |  |  |  |
| *gga-let-7i* | MIMAT0001098 | UGAGGUAGUAGUUUGUGCUGU | 21 |  |  |  |
| *gga-let-7j-5p** | MIMAT0001181 | UGAGGUAGUAGGUUGUAUAGUU | 22 | *hsa-let-7a-5p* | MIMAT0000062 | *bta-let-7a-5, ssa-let-7a-5p, ssc-let-7a* |
| *gga-let-7k-5p* | MIMAT0001182 | UGAGGUAGUAGAUUGAAUAGUU | 22 |  |  | *ssa-let-7e-5p* |
| *gga-miR-9-5p* | MIMAT0001195 | UCUUUGGUUAUCUAGCUGUAUGA | 23 | *hsa-miR-9-5p* | MIMAT0000441 | *ssa-miR-9a-5p* |
| *gga-miR-10a-5p* | MIMAT0007731 | UACCCUGUAGAUCCGAAUUUGU | 22 |  |  | *ssc-miR-10a-5p* |
| *gga-miR-10b-5p* | MIMAT0001148 | UACCCUGUAGAACCGAAUUUGU | 22 |  |  | *ssa-miR-10b-5p, ssc-miR-10b* |
| *gga-miR-16-5p* | MIMAT0001116 | UAGCAGCACGUAAAUAUUGGUG | 22 |  |  | *bta-miR-16a, ssa-miR-16b-5p* |
| *gga-miR-21-5p* | MIMAT0003774 | UAGCUUAUCAGACUGAUGUUGA | 22 | *hsa-miR-21-5p* | MIMAT0000076 | *ssc-miR-21* |
| *gga-miR-22-3p* | MIMAT0007288 | AAGCUGCCAGUUGAAGAACUGU | 22 | *hsa-miR-22-3p* | MIMAT0000077 | *ssc-miR-22-3p* |
| *gga-miR-23b-3p* | MIMAT0001186 | AUCACAUUGCCAGGGAUUACC | 21 | *hsa-miR-23b-3p* | MIMAT0000418 |  |
| *gga-miR-26a-5p* | MIMAT0001118 | UUCAAGUAAUCCAGGAUAGGC | 21 |  |  |  |
| *gga-miR-27b-3p* | MIMAT0001187 | UUCACAGUGGCUAAGUUCUGC | 21 | *hsa-miR-27b-3p* | MIMAT0000419 | *bta-miR-27b, ssa-miR-27b-3p, ssc-miR-27b-3p* |
| *gga-miR-29a-3p* | MIMAT0001096 | UAGCACCAUUUGAAAUCGGUU | 21 |  |  |  |
| *gga-miR-29c-3p* | MIMAT0001183 | UAGCACCAUUUGAAAUCGGU | 20 |  |  |  |
| *gga-miR-30a-5p* | MIMAT0001135 | UGUAAACAUCCUCGACUGGAAG | 22 | *hsa-miR-30a-5p* | MIMAT0000087 | *ssc-miR-30a-5p* |
| *gga-miR-30d* | MIMAT0001129 | UGUAAACAUCCCCGACUGGAAG | 22 | *hsa-miR-30d-5p* | MIMAT0000245 |  |
| *gga-miR-30e-3p* | MIMAT0026538 | UUUCAGUCGGAUGUUUACAGC | 21 |  |  |  |
| *gga-miR-30e-5p* | MIMAT0001177 | UGUAAACAUCCUUGACUGG | 19 |  |  |  |
| *gga-miR-92-3p* | MIMAT0001109 | UAUUGCACUUGUCCCGGCCUG | 21 |  |  |  |
| *gga-miR-99a-5p* | MIMAT0001103 | AACCCGUAGAUCCGAUCUUGUG | 22 | *hsa-miR-99a-5p* | MIMAT0000097 | *ssa-miR-99-5p, ssc-miR-99a* |
| *gga-miR-100-5p* | MIMAT0001178 | AACCCGUAGAUCCGAACUUGUG | 22 |  |  | *bta-miR-100, ssa-miR-100a-5p, ssc-miR-100* |
| *gga-miR-101-3p* | MIMAT0001185 | GUACAGUACUGUGAUAACUGAA | 22 |  |  |  |
| *gga-miR-103-3p* | MIMAT0001145 | AGCAGCAUUGUACAGGGCUAUGA | 23 | *hsa-miR-103a-3p* | MIMAT0000101 | *bta-miR-103, ssa-miR-103-3p, ssc-miR-103* |
| *gga-miR-107-3p* | MIMAT0001147 | AGCAGCAUUGUACAGGGCUAUCA | 23 | *hsa-miR-107* | MIMAT0000104 | *ssc-miR-107* |
| *gga-miR-125b-3p* | MIMAT0026493 | ACAAGUCAGGCUCUUGGGACCU | 22 |  |  |  |
| *gga-miR-125b-5p* | MIMAT0001105 | UCCCUGAGACCCUAACUUGUGA | 22 |  |  | *bta-miR-125b, ssa-miR-125a-5p, ssc-miR-125b* |
| *gga-miR-126-3p* | MIMAT0001169 | UCGUACCGUGAGUAAUAAUGCGC | 23 |  |  |  |
| *gga-miR-126-5p* | MIMAT0003723 | CAUUAUUACUUUUGGUACGCG | 21 |  |  | *bta-miR-126-5p, ssa-miR-126-5p, ssc-miR-126-5p* |
| *gga-miR-130c-3p* | MIMAT0007734 | CAGUGCAAUGUUAAAAGGGCAU | 22 | *hsa-miR-130a-3p* | MIMAT0000425 | *bta-miR-130a, ssc-miR-130a* |
| *gga-miR-133a-3p* | MIMAT0001126 | UUGGUCCCCUUCAACCAGCUGU | 22 |  |  | *ssa-miR-133a-3p* |
| *gga-miR-133c-3p* | MIMAT0001176 | UUGGUCCCCUUCAACCAGCUGC | 22 |  |  |  |
| *gga-miR-140-3p* | MIMAT0003722 | CCACAGGGUAGAACCACGGAC | 21 |  |  |  |
| *gga-miR-142-5p* | MIMAT0001193 | CCCAUAAAGUAGAAAGCACUAC | 22 |  |  |  |
| *gga-miR-146a-5p* | MIMAT0001163 | UGAGAACUGAAUUCCAUGGGUU | 22 | *hsa-miR-146a-5p* | MIMAT0000449 | *ssc-miR-146a-5p* |
| *gga-miR-148a-3p* | MIMAT0001120 | UCAGUGCACUACAGAACUUUGU | 22 | *hsa-miR-148a-3p* | MIMAT0000243 | *bta-miR-148a, ssc-miR-148a-3p* |
| *gga-miR-181a-3p* | MIMAT0001150 | ACCAUCGACCGUUGAUUGUACC | 22 |  |  |  |
| *gga-miR-181a-5p* | MIMAT0001168 | AACAUUCAACGCUGUCGGUGAGU | 23 | *hsa-miR-181a-5p* | MIMAT0000256 | *ssa-miR-181a-5p* |
| *gga-miR-181b-5p* | MIMAT0001151 | AACAUUCAUUGCUGUCGGUGGG | 22 |  |  | *ssa-miR-181c-5p* |
| *gga-miR-183* | MIMAT0001191 | UAUGGCACUGGUAGAAUUCACUG | 23 |  |  | *bta-miR-183, ssc-miR-183* |
| *gga-miR-199-3p* | MIMAT0003721 | UACAGUAGUCUGCACAUUGG | 20 |  |  | *bta-miR-199c* |
| *gga-miR-200a-3p* | MIMAT0001171 | UAACACUGUCUGGUAACGAUGU | 22 | *hsa-miR-200a-3p* | MIMAT0000682 |  |
| *gga-miR-200b-3p* | MIMAT0001172 | UAAUACUGCCUGGUAAUGAUGAU | 23 |  |  | *ssa-miR-200a-3p* |
| *gga-miR-204*** | MIMAT0001156 | UUCCCUUUGUCAUCCUAUGCCU | 22 |  |  | *bta-miR-204, ssa-miR-204-5p, ssc-miR-204* |
| *gga-miR-205a* | MIMAT0001184 | UCCUUCAUUCCACCGGAGUCUG | 22 | *hsa-miR-205-5p* | MIMAT0000266 | *bta-miR-205, ssa-miR-205b-5p, ssc-miR-205* |
| *gga-miR-211*** | MIMAT0003368 | UUCCCUUUGUCAUCCUAUGCCU | 22 | *hsa-let-7a-5p* | MIMAT0000062 | *bta-miR-204, ssa-miR-204-5p, ssc-miR-204* |
| *gga-miR-215-5p* | MIMAT0001134 | AUGACCUAUGAAUUGACAGAC | 21 |  |  | *ssc-miR-215* |
| *gga-miR-221-3p* | MIMAT0001108 | AGCUACAUUGUCUGCUGGGUUUC | 23 |  |  |  |
| *gga-miR-222a* | MIMAT0001107 | AGCUACAUCUGGCUACUGGGUCUC | 24 |  |  | *ssa-miR-222a-3p, ssc-miR-222* |
| *gga-miR-375* | MIMAT0003362 | UUUGUUCGUUCGGCUCGCGUUA | 22 |  |  | *ssa-miR-375-3p* |
| *gga-miR-451* | MIMAT0003775 | AAACCGUUACCAUUACUGAGUUU | 23 |  |  | *bta-miR-451* |
| bta*: Bos taurus,* gga: *Gallus gallus,* hsa: *Homo sapiens,* ssa: *Salmo salar,* ssc: *Sus scrofa.* | | | | | | |
| *, **: Xeno-miRs with the same sequence | | | | | | |
